# Evaluation of the pharmacokinetics, pharmacodynamics, and liver ultrasound imaging characteristics of a novel perfluoropropane microbubble in Chinese volunteers

**DOI:** 10.3389/fphar.2025.1704972

**Published:** 2025-11-07

**Authors:** Pengfei Li, Ping Du, Zhixia Zhao, Fei Jia, Weiyue Yu, Peng Qu, Jiangfan Li, Xuhong Wang, Lihong Liu

**Affiliations:** 1 Department of Pharmacy, Beijing Anding Hospital, Capital Medical University/National Clinical Research Center for Mental Disorders/National Center for Mental Disorders/Beijing Key Laboratory of Mental Disorders, Beijing, China; 2 Phase I Clinical Trial Unit, Beijing Chao-Yang Hospital, Capital Medical University, Beijing, China; 3 National Drug Clinical Trial Center, Beijing Institute of Hepatology, Beijing Youan Hospital, Capital Medical University, Beijing, China; 4 Department of Pharmacy, China-Japan Friendship Hospital, Beijing, China; 5 Phase I Clinical Trial Unit, Beijing Luhe Hospital, Capital Medical University, Beijing, China

**Keywords:** perfluoropropane, microbubble, pharmacokinetics, pharmacodynamics, liverultrasound imaging

## Abstract

**Background and Objective:**

Perfluoropropane (PFP), a lipidbased microbubble preparation, is a novel ultrasound contrast agent. This study aims to evaluate the pharmacokinetics, pharmacodynamics, and liver ultrasound enhancement characteristics of PFP microbubble, a novel ultrasound contrast agent, in healthy Chinese subjects.

**Methods:**

This study is a multicenter, open-label, three-doses, single-dose Phase I clinical trial of the PFP ultrasound contrast agent conducted in healthy male and female volunteers. Screening healthy subjects for inclusion in the trial, with a pre-trial and three dose groups of low (6 μL/kg), medium (12 μL/kg), and high (18 μL/kg), were conducted sequentially from the low-dose group to the high-dose group.

**Results:**

In this study, a total of 146 volunteers were screened, and 39 subjects were enrolled, including 3 participants in the pilot study and 12 participants in each of the low, medium, and high dose groups. No subjects withdrew early from the trial, and all 39 individuals completed the entire trial. After injection, PFP is primarily distributed in the blood and rapidly peaks and disappears in both blood and exhaled gas. Pulmonary excretion is the primary excretion pathway for PFP. The cumulative excretion rates of peak concentration (C_max_), area under the curve (AUC_0-t_ and AUC  0−∞ ), and exhaled gas in blood and exhaled gas do not follow a dose linear relationship due to individual differences; however, the observed average values demonstrate an increasing trend. Significant contrast enhancement was observed after injection of various doses of PFP. With low doses (6 μL/kg), the agent meets the requirements for liver ultrasound diagnosis in this study. Overall, PFP has good safety among healthy Chinese subjects.

**Conclusion:**

This study systematically evaluated the pharmacokinetics and pharmacodynamics of PFP in blood and exhaled breath, as well as the ultrasound characteristics of the liver. We observed for the first time the dynamic changes in blood and exhaled breath between different doses of PFP, and also determined for the first time the appropriate ultrasound diagnostic dose for the experimental formulation. This study presents a valuable methodological framework and reference point for future research in this field.

**Clinical Trial Registration:**

This study was registered on 24 November 2020 at the Chinese Clinical Trial Registry (CTR20202343).

## 1 Introduction

Ultrasound imaging, magnetic resonance imaging (MRI), computed tomography (CT), and positron emission tomography (PET) are commonly used imaging methods in clinical practice. Ultrasound imaging, in particular, offers numerous advantages over other imaging methods (Studwell and Kotton, 2011). It is noninvasive, radiation-free, relatively cost-effective, widely applicable, and suitable for bedside use. Its unparalleled advantage lies in its ability to image soft tissues, particularly for real-time monitoring of treatment processes (Roovers et al., 2019; Klibanov et al., 2010).

Ultrasound contrast agents (UCA) are diagnostic drugs that significantly enhance the detection signal of medical ultrasound diagnosis (Kooiman et al., 2014; Sirsi and Borden, 2009). These agents are preparations containing microbubbles. Gas-encapsulated microbubble preparations have strong ultrasound scattering properties. After intravenous injection into the microcirculation of various organs in the body (Wong et al., 2012; Hudson et al., 2014; Haendl et al., 2010; Min et al., 2017; Eisenbrey et al., 2011), the ultrasound echo signal can be significantly enhanced, leading to a significant improvement in the image quality of tissues and organs, ultimately enhancing the ultrasound diagnostic effect (Segers et al., 2015).

**FIGURE 1:**
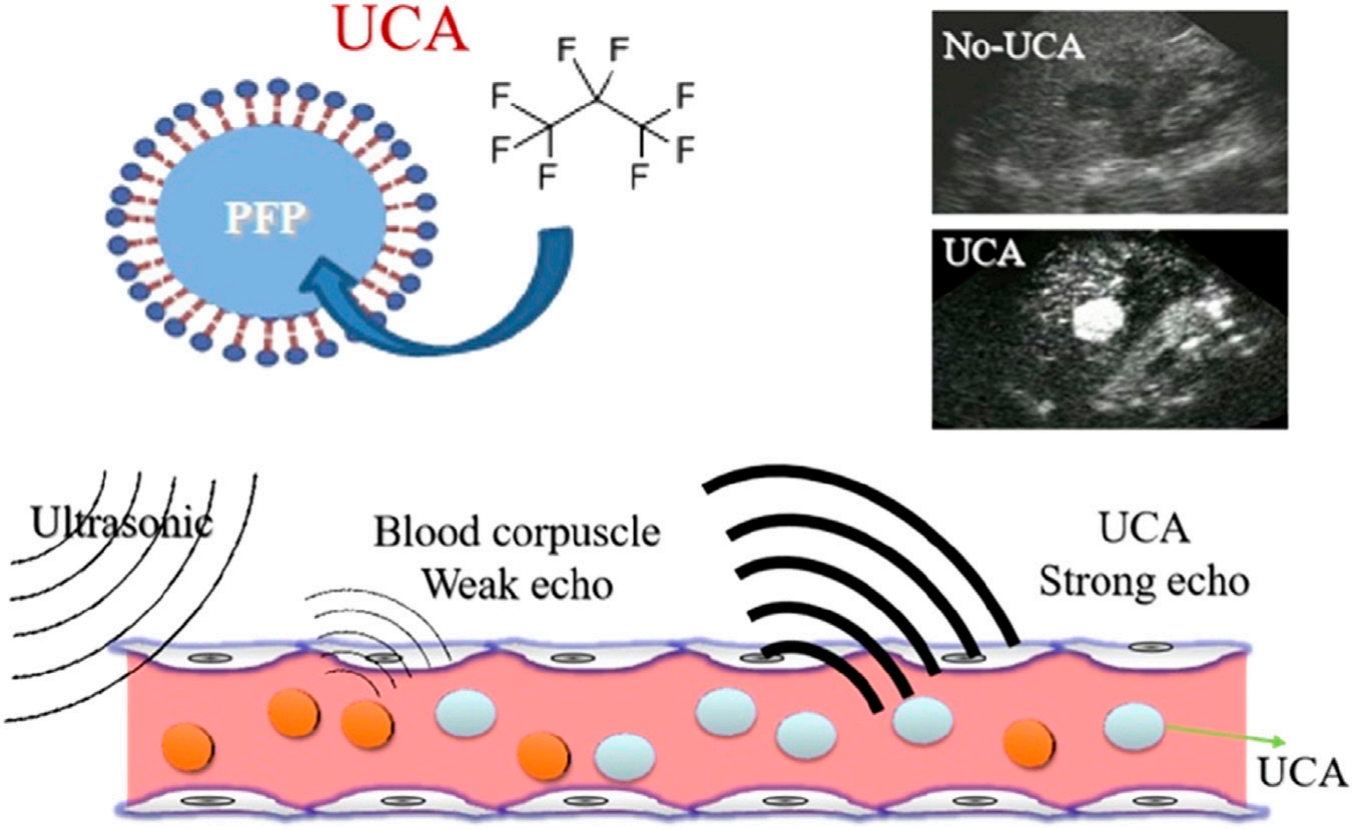
PFP microbubble preparation structure and ultrasound enhancement effect.

Lipid-based microbubble preparations, such as perfluoropropane (PFP) (Li et al., 2024; Yang et al., 2006; Yang et al., 2005) and perfluorobutane (PFB) (Li et al., 2017; Landmark et al., 2008), are novel ultrasound contrast agents (Figure 1). They offer several key advantages (Dietrich et al., 2020; Aziz et al., 2022): a flexible outer shell material that is not easily embedded in microvessels; directional adhesion to tissue cells, leading to longer imaging time; and generally no immune reactions.

There are few reports on the pharmacokinetics of PFP in the human body. Fornage BD directly confirmed that the perfluorocarbon gas (here perfluoropentane) in microbubbles is mainly excreted through respiration, laying the foundation for the pharmacokinetic studies of all subsequent ultrasound contrast agents (Fornage et al., 1993). To date, only we have published studies on the pharmacokinetics and pharmacodynamics of single-dose PFP in the Chinese population (Li et al., 2024). Yang L conducted research on the collection of exhaled breath and the pharmacokinetics of PFP in dogs (Yang et al., 2006; Yang et al., 2005). However, each type of ultrasound microbubble contrast agent exhibits unique *in vivo* behavior due to disparities in microbubble particle size, stiffness, and surface modification. Consequently, it is imperative to investigate the pharmacokinetic differences and ultrasound imaging characteristics of microbubbles from various sources and at different doses. Such research holds significant clinical relevance. This study aims to evaluate the pharmacokinetic characteristics and safety of the novel ultrasound contrast agent PFP microbubbles in healthy Chinese subjects through systematic monitoring. Additionally, we aim to assess the ultrasound enhancement features in the liver.

## 2 Materials and methods

### 2.1 Clinical trial registration and ethical approval

This study strictly adhered to the Good Clinical Practice (GCP) guidelines of the National Medical Products Administration (NMPA) and the ethical principles outlined in the Helsinki Declaration. This study was registered on 24 November 2020 at the Chinese Clinical Trial Registry (CTR20202343). Prior to commencing the study, both the researchers and the sponsors submitted trial-related documents and materials to the ethics committees of Beijing Chaoyang Hospital, affiliated with Capital Medical University, and Beijing Luhe Hospital, also affiliated with Capital Medical University. The ethics committees approved the protocol, informed consent form, subject recruitment materials and procedures, as well as any other written information that needed to be signed and dated for provision to the subjects with the ethical approval number 2019-drug-14-1 and 2021-LHYW-030-01. Researchers ensured that participants were fully and clearly informed about the trial’s purpose, potential risks, and other hazards associated with the clinical study. They also ensured that participants volunteered to participate. Prior to commencing clinical studies, including all screening procedures to identify eligible subjects, a voluntary informed consent form had to be signed by each subject.

### 2.2 Drugs and preparation

The experimental drug used in this study is the injection-grade PFP microbubble (Ttrade name: FRD001), which is a freeze-dried powder injection filled with inert gas in the bottle. Each bottle contains 29 mg of PFP, with a batch number of 20201001, provided by Beijing Feiruida Medical Technology Co., Ltd. The indications for the experimental drug are ultrasound diagnosis of the heart, kidneys, liver, and other organs. Depending on the ultrasound scenario, different dosage options are available. Based on the results of animal efficacy trials, the recommended clinical dosage range for liver ultrasound is 4–12 μL/kg. According to the NMPA guidelines for estimating the maximum recommended initial dose of drugs in the first clinical trial of healthy adult volunteers and acute toxicity test data in rats and dogs, the maximum safe dose of the experimental drug in humans is calculated to be 72 μL/kg and 120 μL/kg, respectively. Therefore, the three doses (6 μL/kg, 12 μL/kg, 18 μL/kg) proposed in this experiment are considered safe. The 18 μL/kg dose is primarily used for further safety assessment.

Preparation of the experimental drug: Take the experimental drug, attach a syringe, and inject 1 mL of physiological saline into the bottle. Then aspirate back the gas from the 1 mL bottle before removing the syringe. Place the contrast agent bottle in the oscillator clamp and shake it mechanically for 45 s. After the shaking is complete, use a syringe to draw the required dose of medication and inject it intravenously into the subject’s body.

### 2.3 Research design and process

This study is a multicenter, open-label, three doses, single-dose Phase I clinical trial of the PFP ultrasound contrast agent conducted in healthy male and female volunteers. For the preliminary trial, three male healthy volunteers were enrolled, with the primary objective of determining suitable blood and exhaled breath collection points for the formal trial. For the formal trial, 36 healthy volunteers (both male and female) were enrolled and divided into three dose groups: low, medium, and high. The experiment was conducted sequentially from the low dose group to the high dose group. Qualified healthy volunteers were selected and admitted to the Phase I ward 1 day prior to the start of the trial. Body weight was measured before administration, vital signs were evaluated, and the volunteers were administered either a low dose (6 μL/kg), medium dose (12 μL/kg), or high dose (18 μL/kg) of the PFP ultrasound contrast agent based on their body weight. Blood samples were collected from the elbow vein within 30 min before administration and at 1, 1.5, 2, 2.5, 3, 4, 5, 6, 7, and 10 min after the start of administration. Exhaled breath was collected for 1 min within 30 min before administration, and all exhaled breath was collected for 30 min after administration (0-2, 2-4, 4-6, 6-8, 8-10, 10-12, 12-14, 14-16, 16-18, 18-20, 20-22, 22-24, 24-26, 2628, 28–30 min). The headspace injection GC-MS method was used to detect the concentration of PFP in both blood and exhaled gas. This allowed for the evaluation of the pharmacokinetic characteristics of the ultrasound contrast agents in healthy volunteers. Simultaneously with collecting biological samples, liver ultrasound examinations were performed. Signals were continuously collected from 5 min prior to administration until 10 min post-administration. Signals were also collected every 2 min for about 10 s from 10 to 20 min and every 4 min for about 10 s from 20 to 40 min to assess the ultrasound enhancement characteristics of the contrast agents in the liver.

### 2.4 Inclusion and exclusion criteria

Inclusion Criteria: Individuals between the ages of 18 and 45 years old. Body mass index (BMI) ranging from 19 to 24 (BMI calculated as weight (kg) divided by the square of height (m^2^)). Normal or abnormal vital signs, physical examination, laboratory examination, chest X-ray, 12-lead electrocardiogram, and other indicators with no clinical significance. Volunteers who are capable and willing to follow the research procedures and provide an informed consent form with their signed name and date.

Exclusion Criteria: Individuals with a history of major illnesses or current major illnesses such as cardiovascular, liver, kidney, digestive, psychiatric, and neurological conditions. Individuals with mental or physical disabilities. Individuals with an allergic constitution or known allergies to the components of the medicine. Alcoholics or individuals testing positive for alcohol. Smokers or individuals testing positive for smoking. History of drug abuse or positive urine drug screening. Use of any prescription drugs or traditional Chinese herbs within 4 weeks prior to enrollment. Screening positive for blood donation or blood loss of ≥200 mL within the previous month.

### 2.5 Safety evaluation

Subjects who withdraw early during the baseline period, during medication, and post-medication should undergo a safety assessment prior to their withdrawal. The safety evaluation indicators include adverse events (AEs) and serious adverse events (SAEs) that occur during treatment, as well as vital signs (systolic and diastolic blood pressure, pulse, body temperature, respiratory rate, and percutaneous blood oxygen saturation), physical examination, 12-lead electrocardiogram (ECG), and clinical laboratory examination (blood routine, urine routine, blood biochemistry, coagulation function).

### 2.6 Pharmacokinetics and statistical methods

Based on the principle of intention to analyze (ITT), all enrolled volunteers will be included in the full analysis set (FAS) of this trial. For volunteers who have not used the investigational drug or have no data after enrollment, they may be excluded from the FAS set. The FAS set is mainly used for demographic, baseline characteristics, and validity analysis. All volunteers who were randomly assigned to the study and administered medication were included in the safety analysis set (SS), which was used for safety analysis. Volunteers who have received the investigational drug and have at least one valid drug concentration data during the trial period are included in the Pharmacokinetic Concentration Analysis Set (PKCS) for descriptive analysis of the drug concentration of the investigational drug. Volunteers who have received the investigational drug and have at least one pharmacokinetic parameter data during the trial period are included in the Pharmacokinetic Parameter Analysis Set (PKPS) for pharmacokinetic parameter analysis of the investigational drug. The division of the above analysis set will be discussed at the data review meeting and finally determined before formal statistical analysis.

Unless otherwise specified, baseline is defined as the visit results of volunteers before medication. If there are multiple visits before medication, the closest visit result to the medication date will be used as baseline data. This analysis is based on available observational data and does not fill in missing data; If there are outliers, the data review meeting will discuss and determine the handling method. Statistical analysis mainly includes analysis of trial completion status, demographic and baseline characteristics, study drug exposure, safety analysis, and pharmacokinetic analysis, with descriptive statistics being the main method of statistical analysis. Unless otherwise specified, all statistical tests will use a two-sided t-test with α = 0.05, and the confidence interval will use a two-sided 95% confidence interval. Based on this, bilateral t-tests (α = 0.05, 95% confidence interval) were used for different groups and genders in this study.

The sample concentrations of each subject at actual sampling time points will be summarized using descriptive statistical methods. Pharmacokinetic (PK) parameters of the drugs will be calculated using the non-compartmental analysis (NCA) model available in SAS version 9.3 and above to comprehensively reflect the characteristics of drug absorption, distribution, metabolism, and excretion in the human body. This analysis will primarily include the determination of peak concentration (C_max_), time to peak concentration (T_max_), area under the curve (AUC_0-t_ and AUC  0−∞ ), elimination half-life (t_1/2_), apparent distribution volume to dose concentration ratio (V_d_/F), and clearance rate to dose concentration ratio (CL/F). Sample size, arithmetic mean, geometric mean, standard deviation, coefficient of variation, median, minimum, and maximum were used to indicate the analysis results of pharmacokinetic parameters. Calculate the expiratory excretion rate of perfluoropropane for volunteers, grouped according to the experimental design, and subsequently perform descriptive analysis on the obtained results.

## 3 Results

### 3.1 Distribution of subjects

**FIGURE 2:**
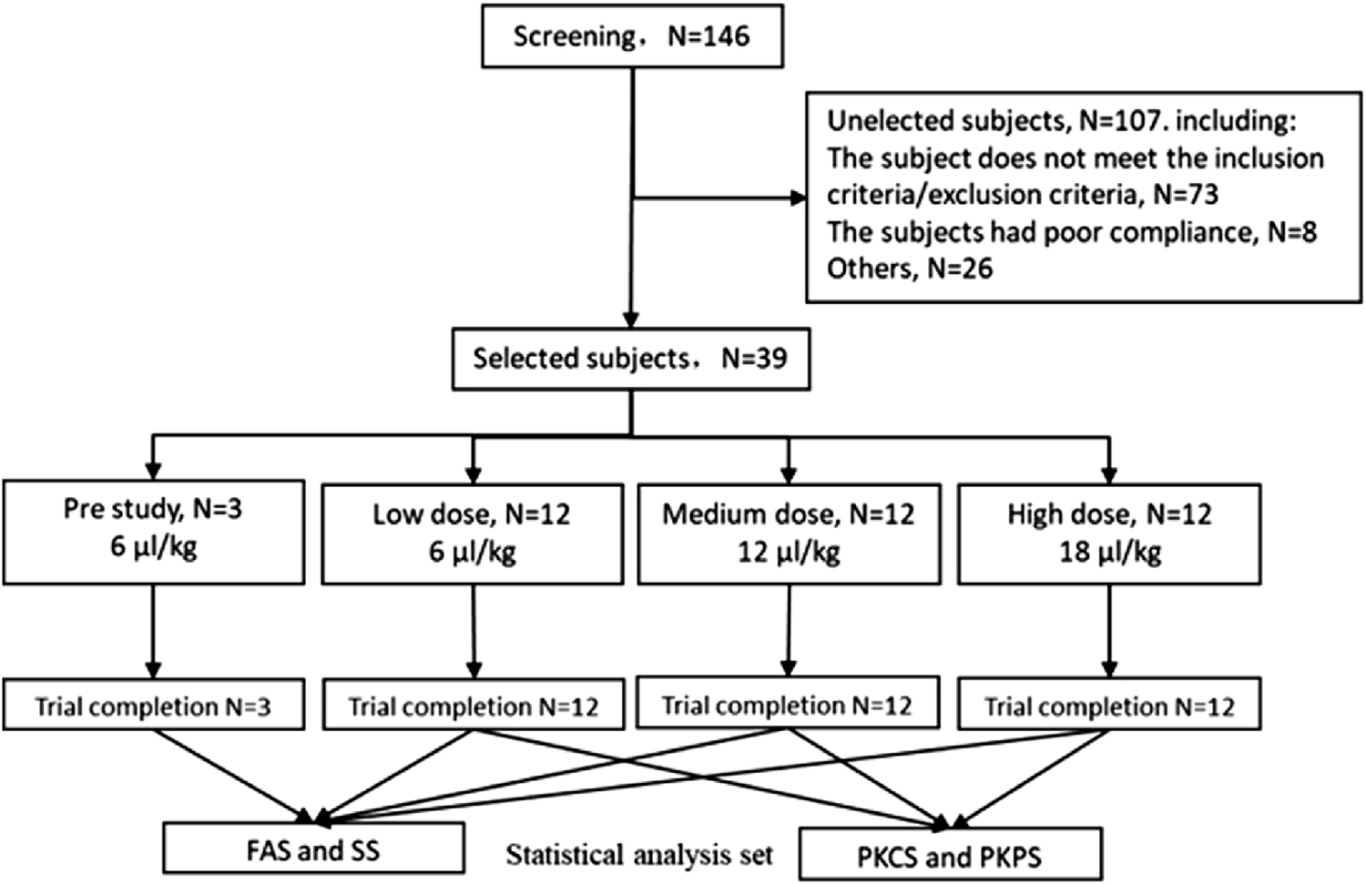
The screening and completion process of volunteers.

In this study, a total of 146 volunteers were screened, and 39 subjects were enrolled, including 3 participants in the pilot study and 12 participants in each of the low, medium, and high dose groups. No subjects withdrew early from the trial, and all 39 individuals completed the entire trial. After the data review meeting, it was confirmed that no subjects were excluded from the analysis population. Ultimately, all 39 subjects were included in the FAS and SS. Except for 3 pre-trial subjects, all 36 subjects were included in the PKCS and PKPS. The distribution of volunteers in this study is shown in Table 1, the screening and completion process of volunteers is shown in Figure 2, and the overview of selected FAS subjects is shown in Table 2. We describe gender and ethnicity based on their entry and exit status to reveal their distribution. We conducted t-tests comparing age, height, weight, and BMI values between three dose groups, and found no significant differences between the different dose groups (*P* > 0.05), indicating that there were no universal demographic differences among the subjects entering each dose group.

**TABLE 1:** Volunteer distribution list for screening, inclusion, and completion of trials.

Trial stage	Subject distribution	Pre study 6 μL/kg	Low dose 6 μL/kg	Medium dose 12 μL/kg	High dose 18 μL/kg	Total
Screening	Screening subjects, n Selected subjects, n (%)	9 3 (33.3)	50 12 (24.0)	39 12 (30.8)	48 12 (25.0)	146 39 (26.7)
	Unelected subjects, n (%)	6 (66.7)	38 (76.0)	27 (69.2)	36 (75.0)	107 (73.3)
Completion	Complete, n (%) Early exit, n (%)	3 (100.0) 0	12 (100.0) 0	12 (100.0) 0	12 (100.0) 0	39 (100.0) 0
Statistical analysis set	FAS, n (%) SS, n (%) PKCS, n (%)	3 (100.0) 3 (100.0) 0	12 (100.0) 12 (100.0) 12 (100.0)	12 (100.0) 12 (100.0) 12 (100.0)	12 (100.0) 12 (100.0) 12 (100.0)	39 (100.0) 39 (100.0) 36 (92.3)
	PKPS, n (%)	0	12 (100.0)	12 (100.0)	12 (100.0)	36 (92.3)

The denominator in the screening situation is the number of screenings, calculated based on the total number of informed signatures. The denominator of the completed experiment and statistical analysis set is the number of participants.

**TABLE 2:** List of general information of FAS subjects.

General information	Features and classification	Pre study 6 μL/kg	Low dose 6 μL/kg	Medium dose 12 μL/kg	High dose 18 μL/kg	Total
Number		3	12	12	12	39
Age	Mean (standard deviation) 18-30, n (%)	32.3 (10.50) 1 (33.3)	30.3 (8.56) 6 (50.0)	27.3 (5.72) 9 (75.0)	30.8 (7.48) 7 (58.3)	29.7 (7.46) 23 (59.0)
	31-45, n (%)	2 (66.7)	6 (50.0)	3 (25.0)	5 (41.7)	16 (41.0)
Height (cm)	Mean (standard deviation)	175.2 (8.81)	168.9 (7.22)	166.3 (7.90)	163.2 (11.19)	166.8 (9.20)
Weight (kg)	Mean (standard deviation)	69.2 (7.92)	64.7 (6.50)	59.2 (6.92)	58.6 (6.23)	61.5 (7.27)
BMI(kg/m^2^)	Mean (standard deviation)	22.4 (1.19)	22.8 (0.83)	21.4 (1.45)	22.1 (1.45)	22.1 (1.33)
Gender	Male, n (%) Female, n (%)	3 (100.0) 0	8 (66.7) 4 (33.3)	5 (41.7) 7 (58.3)	5 (41.7) 7 (58.3)	21 (53.8) 18 (46.2)
Nation	Han ethnicity, n (%) Other ethnicity, n (%)	2 (66.7) 1 (33.3)	12 (100.0) 0	11 (91.7) 1 (8.3)	12 (100.0) 0	37 (94.9) 2 (5.1)

### 3.2 Pharmacokinetic and excretion analysis

**FIGURE 3:**
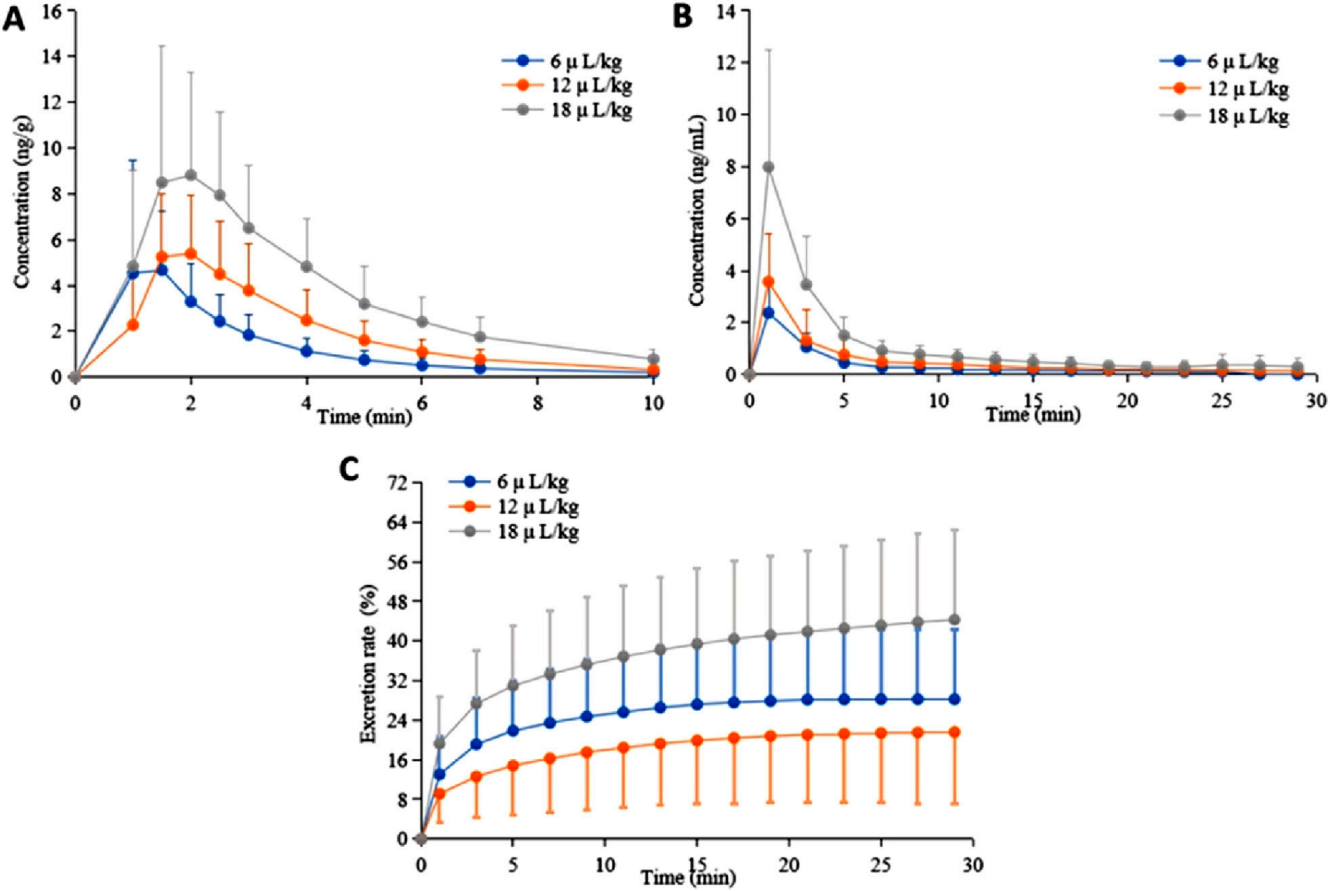
The average concentration time curves of whole blood **(A)** and exhaled gas **(B)** and the cumulative excretion curve of exhaled gas **(C)** of the subjects after administration.

The average blood drug concentration-time curves of each dose group of subjects after administration are presented in Figure 3A, and a list of pharmacokinetic parameters is provided in Table 3. The C_max_ values of PFP in the blood were 1.25 ± 0.26, 1.72 ± 0.40, and 1.83 ± 0.65 min, respectively. The t_1/2_ values were 2.07 ± 0.39, 2.08 ± 0.41, and 2.29 ± 0.26 min, respectively. The CL/F values were 18.68 ± 8.55, 22.37 ± 11.90, and 16.83 ± 10.07 kg/min, respectively. The V_d_/F values were 53.35 ± 20.80, 63.28 ± 26.18, and 58.73 ± 38.42 kg, respectively.

**TABLE 3:** List of main pharmacokinetic parameters in blood and exhaled breath of subjects after administration.

Whole blood	Dose (μL/kg)	C_max_ (ng/g)	T_max_ (min)	AUC_0-t_ (min^a^ng/g)	AUC 0−∞ (min^a^ng/g)	t_1/2_ (min)	CL/F (kg/min)	V_d_/F (kg)
	6	5.78 ± 4.17	1.25 ± 0.26^a^	12.53 ± 7.46	13.14 ± 7.65	2.07 ± 0.39	18.68 ± 8.55	53.35 ± 20.80
	12	6.06 ± 2.77	1.72 ± 0.40	19.13 ± 8.84	20.26 ± 9.26^a^	2.08 ± 0.41	22.37 ± 11.90	63.28 ± 26.18
	18	10.37 ± 4.95	1.83 ± 0.65	36.15 ± 16.30	39.86 ± 17.88	2.29 ± 0.26	16.83 ± 10.07	58.73 ± 38.42
Exhaled breatd	Dose (μL/kg)	C_max_ (ng/mL)	T_max_ (min)	AUC_0-t_ (min^a^ng/mL)	AUC 0−∞ (min^a^ng/mL)	t_1/2_ (min)	CL/F (L/min)	V_d_/F (L)
	6	2.30 ± 1.13	1.00 ± 0.00	8.35 ± 4.60^a^	10.00 ± 5.02^a^	6.56 ± 2.35	26.04 ± 15.77	260.32 ± 137.92
	12	3.31 ± 2.00^a^	1.17 ± 0.58	13.42 ± 9.63^a^	17.11 ± 9.35^a^	7.69 ± 3.06	32.01 ± 20.15	353.39 ± 249.62
	18	8.41 ± 4.10	1.17 ± 0.58	32.59 ± 16.40	35.77 ± 19.84	7.80 ± 1.69	18.71 ± 9.79	205.18 ± 107.07

^a^ There is a significant difference in pharmacokinetic parameters between genders, and the values of the labeled pharmacokinetic parameters for females are significantly lower than those for males.

The average exhaled drug concentration-time curves of each dose group of subjects after administration are presented in Figure 3B, and a list of pharmacokinetic parameters is provided in Table 3. The C_max_ values of PFP in exhaled breath were 1.00 ± 0.00, 1.17 ± 0.58, and 1.17 ± 0.58 min, respectively. The t_1/2_ values were 6.56 ± 2.35, 7.69 ± 3.06, and 7.80 ± 1.69 min, respectively. The CL/F values were 26.04 ± 15.77, 32.01 ± 20.15, and 18.71 ± 9.79 L/min, respectively. The V_d_/F values were 260.32 ± 137.92, 353.39 ± 249.62, and 205.18 ± 107.07 L, respectively.

30 min after administration, the cumulative excretion curves of PFP in the exhaled breath of each dose group of subjects are presented in Figure 3C. The low dose group has a cumulative excretion rate of 28.23% ± 14.02% (male: 32.66% ± 14.44% vs. female: 19.36% ± 8.79%, *p* = 0.078), the medium dose group has a rate of 21.61% ± 14.41% (male: 33.97% ± 9.61% vs. female: 12.77% ± 10.04%, *p* = 0.005), and the high dose group has a rate of 44.27% ± 18.19% (male: 53.39% ± 15.85% vs. female: 37.75% ± 17.88%, *p* = 0.143).

### 3.3 Contrast-enhanced ultrasound analysis

**FIGURE 4:**
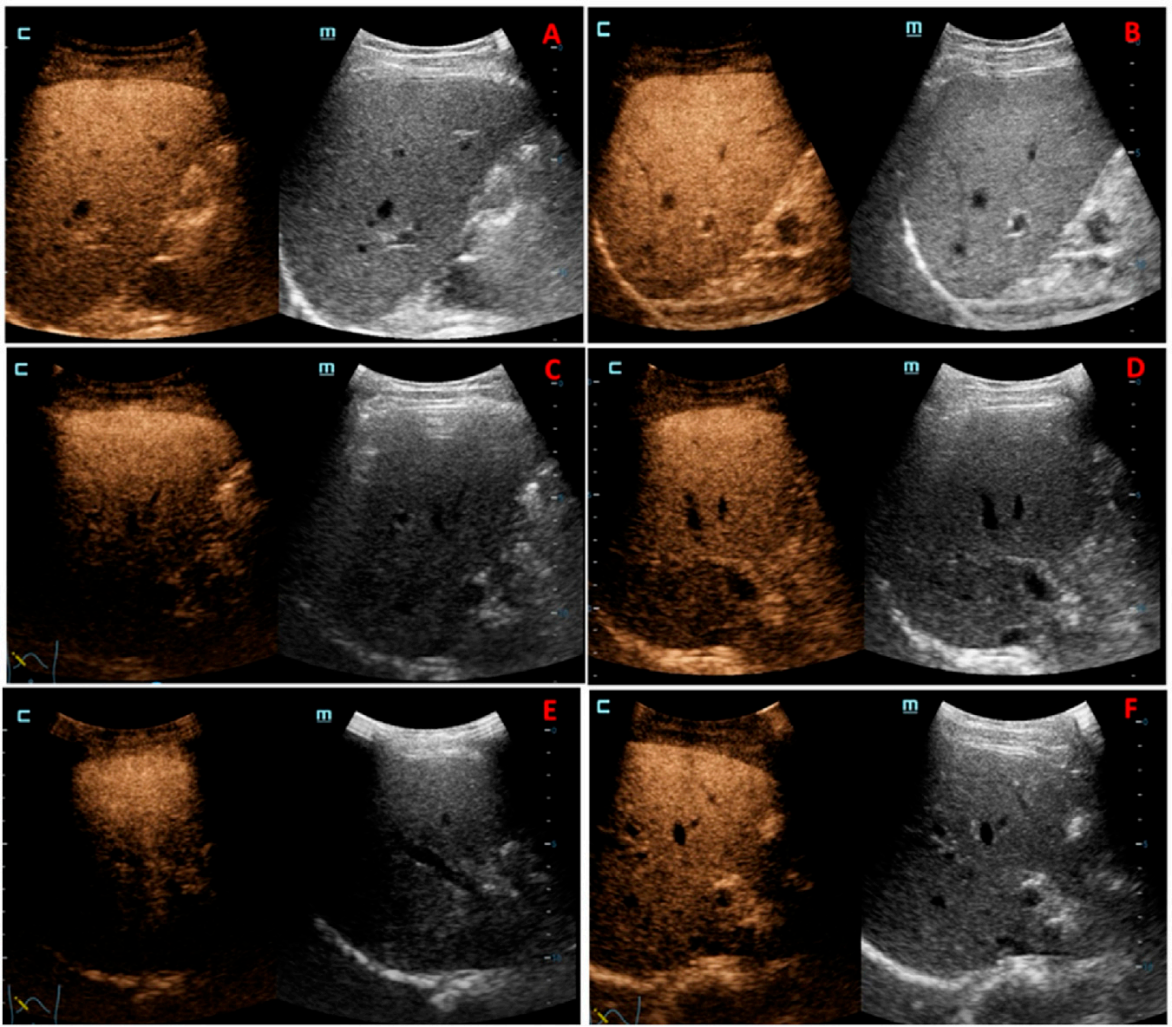
Ultrasound images at 5 min **(A,C,E)** and 20 min **(B,D,F)** after administration in the low-dose group, medium dose group, and high-dose group.

In this study, the average peak intensity and peak time of the TIC curve of low, medium, and high dose ultrasound images were 46.15 ± 5.86 dB and 52.03 ± 6.89 s, 44.32 ± 8.51 dB and 67.14 ± 16.55 s, 44.71 ± 7.02 dB and 67.33 ± 14.14 s, respectively. After the administration of PFP microbubbles for injection, significant contrast enhancement was observed, with clear post-vascular (Kupffer phase) enhancement (>20 min), as shown in Figures 4A,C,E. When compared to the low-dose group, the deep echogenicity of ultrasound images in the medium and high-dose groups was significantly reduced, and there was varying degrees of doserelated overattenuation. The ultrasound images in the medium dose group showed overattenuation of >50%, while those in the high-dose group showed overattenuation of >90%, as shown in Figures 4B,D,F.

### 3.4 Security analysis

In this study, a total of 13 AEs occurred, as shown in Table 4. In the low-dose group, two subjects experienced a total of two treatment-emergent adverse events (TEAEs), all of which were treatment-related, with an incidence rate of 16.7%. No adverse reactions were reported. In the medium-dose group, after administration, five subjects experienced a total of six TEAEs, all of which were treatment-related, with an incidence rate of 41.7%. Two subjects each experienced three adverse reactions, with an incidence rate of 16.7%. In the high-dose group, after administration, five subjects experienced a total of five TEAEs, all of which were treatment-related, with an incidence rate of 41.7%. Four subjects each experienced four adverse reactions, with an incidence rate of 33.3%. All TEAEs were grade 1 in severity; no SAEs occurred; no subjects withdrew from the trial; and all symptoms resolved without treatment. A total of seven TEAEs (19%) that may be related to the study drug occurred in each dose group. In the medium-dose group, two cases had a decrease in globulin and one case had a decrease in white blood cells. In the high-dose group, three cases had an increase in blood bilirubin and one case had discomfort in the stomach, all of which were grade 1 in severity. The symptoms resolved without treatment. Overall safety was good, with no SAEs, no TEAEs leading to withdrawal, and no deaths reported.

**TABLE 4:** List of adverse events.

Number	Dose group	Age	Gender	AE name	Outcome	Grading	Measures taken	Relationship with drugs	SAE?	Withdraw trials?
1	6 μL/kg	42	Female	Menstrual disorders	Symptoms disappear	I	None	May not be relevant	No	No
2	6 μL/kg	26	Female	Total protein decreased	Symptoms disappear	I	None	May not be relevant	No	No
3	12 μL/kg	29	Female	Anemia	Symptoms disappear	I	None	May not be relevant	No	No
4	12 μL/kg	29	Male	Low hemoglobin levels	Symptoms disappear	I	None	Perhaps related	No	No
5	12 μL/kg	27	Female	High white blood cell count in urine	Symptoms disappear	I	None	May not be relevant	No	No
6	12 μL/kg	34	Female	Anemia	Symptoms disappear	I	None	May not be relevant	No	No
7	12 μL/kg	23	Male	Low white blood cell count	Symptoms disappear	I	None	Perhaps related	No	No
8	12 μL/kg	23	Male	Low hemoglobin levels	Symptoms disappear	I	None	Perhaps related	No	No
9	18 μL/kg	21	Male	Elevated blood bilirubin	Symptoms disappear	I	None	Perhaps related	No	No
10	18 μL/kg	32	Female	Anemia	Symptoms disappear	I	None	May not be relevant	No	No
11	18 μL/kg	26	Male	Elevated blood bilirubin	Symptoms disappear	I	None	Perhaps related	No	No
12	18 μL/kg	31	Female	Elevated blood bilirubin	Symptoms disappear	I	None	Perhaps related	No	No
13	18 μL/kg	29	Female	Stomach discomfort	Symptoms disappear	I	None	Perhaps related	No	No

## 4 Discussions

After injection, perfluoropropane is mainly distributed in the blood, reaching peak concentration rapidly and being eliminated quickly. Lung excretion is the primary excretion pathway of perfluoropropane, which reaches peak levels and is rapidly eliminated after injection. However, the exhaled breath t_1/2_ is greater than three times the t_1/2_ in the blood (6.56 ± 2.35, 7.69 ± 3.06, 7.80 ± 1.69 min vs. 2.07 ± 0.39, 2.08 ± 0.41, 2.29 ± 0.26 min). It is speculated that the decrease in PFP concentration in exhaled breath lags behind that in the blood. It is also not ruled out that the calculation deviation may be caused by the blood drug concentration being lower than the lower limit of quantification of the detection method after 10 min of administration. V_d_ does not represent the true volume with physiological significance, but rather the theoretical volume that foreign compounds should occupy based on the measured concentration. The reason why exhaled gas V_d_/F is much larger than blood V_d_/F is mainly due to differences in relative reference (blood vs. exhaled gas) and unit (g vs. mL).

With the increase in dosage, the C_max_, AUC_0-t_, and AUC  0−∞ in the blood and exhaled breath do not conform to a dose-linear relationship due to significant individual differences and other factors, but the observed mean values show an increasing trend. For the pharmacokinetic parameters, the AUC_%Extrap_= (1-AUC_0-t_/AUC  0−∞ ) * 100%) was calculated for the average values of all subjects in the trial. The average AUC_%Extrap_ in the blood and exhaled breath were found to be 6.72% ± 4.05% and 11.35% ± 8.68%, indicating that more than 93% of PFP has been detected in the blood within 10 min and more than 88% of PFP has been detected in exhaled breath within 30 min. Within 10 min, more than 93% of PFP in the blood: This indicates that PFP enters the human body through intravenous injection, and the efficiency of the circulatory system is very high. It quickly spreads throughout the body’s vascular lumen (including microcirculation), which is the basis for its ability to serve as an efficient ultrasound contrast agent - rapidly generating and enhancing ultrasound signals. Within 30 min, more than 88% of PFP was detected in exhaled breath, indicating an extremely rapid and direct clearance process of PFP. It spreads from the bloodstream to the alveoli and is then expelled from the body with respiration. 93% detected in the blood → represents’ input amount ', and 88% detected in exhaled breath → represents’ recovery amount’. The high consistency between the input and recovery amounts (considering experimental errors and the possibility of very small amounts being excreted through other pathways) strongly indicates that PFP is rapidly and completely cleared in its original form through the lungs without any metabolism in the body. If PFP is metabolized by organs such as the liver in the body, its recovery rate in exhaled breath will be much lower than its detection rate in the blood. Because metabolism converts it into other substances (metabolites), which may be excreted through urine or bile instead of being exhaled from the lungs in their original form. The rapid “in out” mode and non metabolic characteristics indicate that PFP has excellent biosafety and tolerability.

The cumulative excretion rate of PFP in exhaled breath did not follow a dose-linear relationship. The average cumulative excretion rate in the medium dose group was lower than that in the low dose group, but the difference was not significant (21.61% ± 14.41% vs. 28.23% ± 14.02%, p = 0.266). Although the cumulative excretion rate of PFP was significantly lower in female subjects than in male subjects only in the medium dose group (female: 12.77% ± 10.04% vs. male: 33.97% ± 9.61%, p = 0.005), the mean values of female subjects were lower than those of male subjects in all three dose groups, and when all subjects in the three groups were compared together, the cumulative excretion rate of PFP in female subjects was significantly lower than that in male subjects (23.95% ± 17.23% vs. 38.78% ± 15.94%, p = 0.011). This is mainly due to the large individual differences, and it is speculated that it may also be related to the larger number of female subjects in the medium and high dose groups (7 female subjects in the medium and high dose groups vs. 4 female subjects in the low dose group, with lower body weight and lower lung expiratory volume). The gender differences observed in this study are speculated to be related to differences in body weight and lung physiology based on their physiological basis. PFP is a contrast agent in the blood pool, and its initial distribution volume is related to body weight (especially blood volume). Female subjects had a lower average body weight, resulting in a higher initial concentration of PFP per unit volume of blood at the same dosage. In cases where lung ventilation capacity is similar or slightly weaker, clearing the same proportion of medication may take longer, which may lead to a decrease in cumulative excretion rate at specific time points. In addition, although there are individual differences, women’s average lung capacity and minute ventilation are usually lower than men’s. PFP is completely diffused through the alveolar membrane and expelled with exhalation, so the relatively low lung ventilation may become a limiting factor for gas expulsion, thereby slowing down the clearance rate. The cumulative excretion rate of PFP in exhaled breath of each dose group does not exceed 50% of the administered dose, which is speculated to be related to the natural rupture of microbubble preparations containing PFP gas in the body, ultrasound-induced rupture, delayed excretion, and below the quantification limit.

Compared to our previous studies (Li et al., 2024), the perfluoropropane microbubble formulation FRD001 in this study maintains the characteristics of rapid peak reaching and rapid elimination in the blood, with a faster peak reaching (T_max_: 1.25 ± 0.26, 1.72 ± 0.40, 1.83 ± 0.65 min vs. 2.06 ± 0.454 min) and slightly slower elimination (t_1/2_ 2.07 ± 0.39, 2.08 ± 0.41, 2.29 ± 0.26 min vs. 1.68 ± 0.326 min). This is mainly due to the slightly larger average particle size distribution of the microbubble formulation in this study, which aligns with the pharmacokinetic and ultrasound imaging results of this formulation in animal experiments. The different measurement methods used in the two studies (ng/g vs. uL/mL) make it difficult to directly compare the pharmacokinetic parameters involved in C_max_, AUC, CL, and V_d_. Overall, there is still a difference of about 50% between individuals, and no gender difference is observed in the blood. However, there are still significant differences in exhalation and excretion, which align with the pharmacokinetic characteristics of this type of drug. This study, along with previous PFB and PFP human studies (Li et al., 2024; Li et al., 2017; Landmark et al., 2008), did not result in significant clinical changes in laboratory tests, vital signs, blood oxygen saturation, or electrocardiograms. Both studies demonstrated good human safety. The pulmonary excretion of PFP microbubbles is not a simple “filtration” process, but an active and staged “gas dissociation and diffusion” process. The core driving force is the high solubility of PFP gas in the blood and the huge gas pressure difference between the alveolar cavity and the blood (Leen and McArdle, 1996). We cannot simply say ‘the lungs filtered out microbubbles’. A more accurate mechanism is that the lungs utilize their inherent gas exchange function to clear PFP gas dissolved in the blood. Microbubbles themselves are just gas phase reservoirs, which supply blood through dissolution (natural process or ultrasound accelerated rupture), and then unload blood to alveoli through differential pressure. In imaging and treatment, ultrasound rupture of microbubbles not only generates signals or delivers drugs, but also significantly alters the pharmacokinetics of PFP, making its clearance in the lungs more rapid and intense.

The excessive attenuation caused by higher doses of PFP can seriously impair the diagnostic ability of ultrasound and indicate that its use has exceeded the ideal imaging window. This dose related excessive attenuation is direct evidence of the saturation effect of contrast agents on microvessels or tissues, where sound waves cannot penetrate the thick microbubble “cloud wall” ahead. At medium and high doses, deep tissues are obscured by “acoustic shadows” and cannot be observed, leading to a significantly increased risk of missed diagnosis (such as the inability to detect small tumors or lesions in the deep). This phenomenon indicates the existence of an optimal diagnostic dose window (low-dose group), beyond which the image quality does not improve but rather decreases. Therefore, medium and high doses are usually not advisable in clinical practice. For PFP contrast agents, the observed excessive attenuation is a key dose limiting signal that warns clinicians to use the lowest effective dose (i.e., low dose) that meets diagnostic requirements to avoid producing acoustic shadows and ensure clear visualization of deep tissue structures, thereby ensuring diagnostic accuracy and safety. Based on the above results and discussions, a low dose of 6 μL/kg can meet the requirements for liver ultrasound diagnosis in this study, the recommended clinical dose range for PFP microbubbles is 4–8 μL/kg (6 ± 2 μL/kg), depending on the depth of the ultrasound site. For instance, in rapid ultrasound of the thyroid, lymph, breast, and blood vessels, a recommended dosage of 4 μL/kg of PFP microbubbles is recommended; The recommended dosage of PFP microbubbles in abdominal (heart, liver, kidney, pancreas, spleen, gallbladder) and deep solid tumors ultrasound is 6 or 8 μL/kg.

Since this study was not a bioequivalence evaluation, the statistical analysis was mainly based on descriptive statistics, and did not conduct a more in-depth and precise evaluation of the sample size or power. Based on previous experience and considering that the coefficient of variation (CV) values of the main pharmacokinetic parameters such as C_max_, AUC_0-t_, and AUC  0−∞ in this study were close to or greater than 50%, the sample size in each dose group of this study was too small, which was a deficiency of this study.

In recent years, researchers have been committed to designing intelligent responsive materials (such as ultrasound responsive nanoparticles) and combining them with the spatiotemporal control ability of external ultrasound to achieve precise release of drugs or genes at specific times and locations, thereby maximizing drug efficacy and reducing side effects (Liang et al., 2025). The *in vivo* process study of ultrasound contrast agents provides a certain foundation for the research of such drugs. In this study, the average particle size of activated PFP was about 1.5–2.5 μm (compared to marketed PFP of 1.1–2.5 μm), and the average microbubble concentration was 1–10*10^9^/mL (compared to marketed PFP of 6.4*10^7^–1.2 *10^10^/mL). The average particle size of microbubbles was comparable to marketed PFP, and the distribution range was more concentrated. The microbubble concentration range was much better than marketed PFP, which fundamentally determined that microbubbles in this study had better contrast effects in the human body at low doses, and the images lasted for more than 20 min.

## 5 Conclusion

In this study, we comprehensively examined the process and safety of a novel ultrasound contrast agent, PFP microbubbles, in healthy Chinese subjects. We also evaluated its contrast effect. After injection, PFP is primarily distributed in the blood and rapidly peaks and disappears in both blood and exhaled gas. Pulmonary excretion is the primary excretion pathway for PFP. The cumulative excretion rates of C_max_, AUC_0-t,_ AUC  0−∞ , and exhaled gas in blood and exhaled gas do not follow a dose linear relationship due to individual differences; however, the observed average values demonstrate an increasing trend. Significant contrast enhancement was observed after injection of various doses of PFP. Furthermore, our findings demonstrate that PFP exhibits an excellent safety profile in healthy Chinese participants, with a dosage of 6 μL/kg proving to be optimal for liver ultrasound diagnostic applications.

## Data Availability

The original contributions presented in the study are included in the article/supplementary material, further inquiries can be directed to the corresponding authors.
